# Macrophage Heterogeneity in the Intestinal Cells of Salmon: Hints From Transcriptomic and Imaging Data

**DOI:** 10.3389/fimmu.2021.798156

**Published:** 2021-12-23

**Authors:** Youngjin Park, Qirui Zhang, Jorge M. O. Fernandes, Geert F. Wiegertjes, Viswanath Kiron

**Affiliations:** ^1^ Faculty of Biosciences and Aquaculture, Nord University, Bodø, Norway; ^2^ Aquaculture and Fisheries Group, Wageningen University & Research, Wageningen, Netherlands

**Keywords:** adherent cells, intestinal cells, macrophages, RNA-seq, miRNAs, Atlantic salmon

## Abstract

The intestine has many types of cells that are present mostly in the epithelium and lamina propria. The importance of the intestinal cells for the mammalian mucosal immune system is well-established. However, there is no in-depth information about many of the intestinal cells in teleosts. In our previous study, we reported that adherent intestinal cells (AIC) predominantly express macrophage-related genes. To gather further evidence that AIC include macrophage-like cells, we compared their phagocytic activity and morphology with those of adherent head kidney cells (AKC), previously characterized as macrophage-like cells. We also compared equally abundant as well as differentially expressed mRNAs and miRNAs between AIC and AKC. AIC had lower phagocytic activity and were larger and more circular than macrophage-like AKC. RNA-Seq data revealed that there were 18309 mRNAs, with 59 miRNAs that were equally abundant between AIC and AKC. Integrative analysis of the mRNA and miRNA transcriptomes revealed macrophage heterogeneity in both AIC and AKC. In addition, analysis of AIC and AKC transcriptomes revealed functional characteristics of mucosal and systemic macrophages. Five pairs with significant negative correlations between miRNA and mRNAs were linked to macrophages and epithelial cells and their interaction could be pointing to macrophage activation and differentiation. The potential macrophage markers suggested in this study should be investigated under different immune conditions to understand the exact macrophage phenotypes.

## Introduction

Intestine is the largest interface between the external environment and the host. Hence, this organ has many immune cells that support the defence system. Fish intestine consists of various immune cells such as macrophages, dendritic-like cells, B and T cells, and intraepithelial lymphocytes; they are present either between the epithelial cells or in the lamina propria of the intestine (1). These cells are assumed to work together as a mucosal barrier, supporting immune responses in the intestine (2).

Cell adhesion is one of the inherent functions of intestinal cells. This fundamental characteristic is essential for both, stimulating cell-cell communication and sustaining tissue structure (3). This ability of cells to adhere to one another can be exploited to obtain a highly enriched cell population for research purposes. For example, in mammals, this characteristic has been used to study adherent cell types such as macrophages (4), epithelial cells (5) and endothelial cells (6). In fish, the head kidney is a key immune organ and we previously suggested that adherent cells from this organ of Atlantic salmon (*Salmo salar*) could include macrophages (7). We also reported that adherent cells from the intestine of Atlantic salmon express a number of genes typical of macrophages and that these cells have phagocytic ability (8), suggesting that AIC comprise macrophages.

All cells including macrophages express messenger RNAs (mRNAs) and microRNAs (miRNAs). MicroRNAs are a group of small RNAs that are able to bind to the 3’ UTR of messenger RNAs and these small RNAs negatively regulate target transcripts by silencing mRNA translation or promoting their degradation (9). In mammals, several studies revealed negative correlations between the expression of miRNA and mRNA, based on the fact that up/downregulated miRNAs give rise to down/upregulated mRNA (10, 11). Other studies addressed miRNA-mRNA interactions to understand the miRNA targeting specificity in mice (12) and insects (13). In fish, a recent study profiled the miRNAs from different tissues and developmental stages of Atlantic salmon (14). Furthermore, Smith, Christian (15) reported that miRNA expressions in adherent head kidney cells could influence macrophage differentiation. However, corresponding information associated with fish intestine do not exist.

Macrophages can display different activation states that reflect different phenotypes acquired in response to distinct environmental signals. Macrophage heterogeneity can be influenced by dietary components, microbiota and cytokines (16–19). For humans, it has also been reported that the local environment of the intestine could imprint specific phenotypes and functions of macrophages (20). In addition, self-replenishment of tissue-resident macrophages is considered a conserved process (21). Considering these characteristics of macrophages, we assume that intestinal macrophages of fish could have unique phenotypes different from those of systemic organs like head kidney. For mammals, it is known that miRNAs have roles in both differentiation of intestinal epithelial cells, dendritic cells, and macrophages (22–24). Fish M1 and M2-like macrophages are believed to produce antimicrobials (nitric oxide and pro-inflammatory cytokines)(M1) or molecules associated with tissue regeneration (arginase and anti-inflammatory cytokines)(M2) similar to their mammalian counterparts (19, 25, 26). Although we reported the presence of macrophages in adherent intestinal cells in our previous study (8), we could not ascertain conclusively the macrophage heterogeneity.

Here, we investigated both mRNA and miRNA transcriptomes in adherent cells from the intestine (AIC) and the head kidney (AKC) of Atlantic salmon. We examined (1) equally abundant macrophage-related mRNAs and miRNAs in AIC and AKC, (2) differentially expressed mRNAs (DEGs) and miRNAs (DE miRNAs) in AIC compared to AKC, (3) negative correlations between DE miRNA and DE target mRNAs, and (4) enriched pathways and GO terms of transcriptomes of AIC and AKC. We discuss the gene profiles of the adherent cells to reveal the presence of heterogeneous macrophage phenotypes. A better knowledge of different cell types in Atlantic salmon is important, both from an evolutionary and fish health perspective.

## Methods

### Ethics Statement

The present study was approved by the National Animal Research Authority in Norway (Mattilsynet; FOTS ID 10050) and all the protocols were according to its guidelines.

### Experimental Animal and Sample Collection

In this study, Atlantic salmon (*Salmo salar*) post smolts were purchased from a commercial producer (Sundsfjord Smolt, Nygårdsjøen, Norway). These fish were raised in a flow-through sea water system (temperature: 7-8°C, dissolved oxygen saturation: 87-92%, 24-h light cycle) at the Research Station of Nord University, Bodø, Norway, and were daily fed a commercial feed (Ewos Micro, Ewos AS, Bergen, Norway) at 1.2% of their body weight. Samples were collected from fish (n = 6) of the weight range 510-590 g. They were starved for 24 h and sacrificed with an overdose of tricaine methane sulphonate (Argent Chemical Laboratories, Redmond, USA; 200 mg/L). Then, distal intestine (DI) and head kidney (HK) were dissected under sterile conditions to isolate the cells from these organs.

### Cell Isolation and Culture

Cells from DI and HK were harvested and grown at 12°C in Leibovitz’s L-15 Medium (L-15; Sigma, Oslo, Norway) as described previously by Park, Zhang (8). Briefly, the isolated DI or HK leukocytes were allowed to adhere on a cell culture dish (Nunc EasYDish, Thermo Fisher Scientific, Oslo, Norway) with 2 mL L-15+ (L-15 medium with 50 U/mL penicillin, 50 μg/mL streptomycin, 2% fetal bovine serum and 10 U/mL heparin) for 2 days at 12°C. Thereafter the medium was removed and the adherent cells on the culture dish were detached by washing three times with 1.5 mL ice-cold PBS (Sigma) supplemented with 5 mM EDTA (Sigma). The cells were centrifuged (500 × *g*, 5 min, 4°C) and re-suspended with 2 mL L-15+. Then, the adherent intestinal cells or adherent head kidney cells were counted using a portable cell counter (Scepter™ 2.0 cell counter, EMD Millipore, Darmstadt, Germany) for further analysis.

### Phagocytosis Assay

First, we assessed the morphological differences of the adherent cells from the AIC and AKC populations. Then we evaluated the phagocytic activities of these cells using phagocytosis assay employing ImageStream^®^X Mk II Imaging Flow Cytometer (Luminex Corporation, Austin, TX, United States). The assay and image analyses were performed based on our previous protocols (7, 8). Briefly, fluorescent bio-particles (pHrodo™ Red *Escherichia coli* Bioparticles, Thermo Fisher Scientific) were added into aliquots containing 0.5 × 10^5^ cells in 100 μL L-15 + at a cell and particle ratio of 1:5 for incubating the cells for 2 h at 12°C. After incubation, the cells with the engulfed particles were washed with 500 μL PBS by centrifugation (500 × g, 5 min, 4°C) and resuspended in 50 μL PBS. Then, we added 1 μL of propidium iodide (PI) to the tubes containing the cells, right before loading into the imaging flow cytometer, to stain dead cells as well as to study the shape of nuclei of macrophage-like cells. Thereafter, more than 10,000 cell images were acquired. During data analysis, the dead cells were excluded by gating the PI positive cell area, and only live cells were considered for the phagocytic assay. We analysed the cell images by adopting the masking strategy and employing the features provided in IDEAS 6.1.822.0 software (Luminex); compared diameter, circularity, and elongation of phagocytes in the AIC and AKC populations. The percent of phagocytosis of bio-particles by macrophages is calculated based on the number of macrophages engulfing the particles among total macrophages.

### mRNA and small RNA Profiling

We performed mRNA and small RNA sequencing to profile their expression in the AIC and AKC (reference cell type). Six biological replicates were used for the study.

### RNA Isolation

Total RNA was extracted from AIC or AKC (500,000 cells) using PicoPure RNA isolation kit (Thermo Fisher Scientific) according to the manufacturer’s protocol. The quality and quantity of the isolated total RNA were assessed using Agilent RNA high sensitivity screen tape kits and Bioanalyzer 2200 TapeStation system (Agilent Technologies, Santa Clara, CA, USA).

### Library Preparation and Illumina Sequencing

For mRNA sequencing, high quality RNA (RNA Integrity Number > 8) from each sample (50 ng) was used for library construction using the NEBNext Ultra II Directional RNA library preparation kit with poly (A) mRNA magnetic isolation module (NEB #E7490; New England BioLabs^®^, Herts, UK) as described previously by Park, Zhang (8). The quality and quantity of sequencing-ready libraries were examined using Agilent DNA high sensitivity screen tape kits and Bioanalyzer 2200 TapeStation system. These individual libraries were pooled at equimolar ratio and sequenced on a NextSeq 500 sequencer (Illumina, San Diego, CA, USA) with a high throughput flow cell (single‐end, 75 bp) at the sequencing facility of Nord University, Bodø, Norway.

A portion of the RNA extracted for the mRNA sequencing was also used for the miRNA sequencing; 50 ng was used for library preparation using the NEXTflex Small RNA-Seq Kit v3 (Bioo Scientific, Austin, TX, USA), following the manufacturer’s protocol. Briefly, RNA was ligated with the NEXTflex 3’ 4N and 5’ 4N Adenylated adapters. Then, the RNA was reverse transcribed into first-strand cDNA and amplified with NEXTflex universal and barcoded primers on a thermocycler (Applied Biosystem, NY, USA) for 25 cycles. The conditions for each cycle were: 95°C for 2 min; 95°C for 20 s, 60°C for 30 s, 72°C for 15 s; 72°C for 2 min. For PAGE size selection, 5 µL of 6X gel loading dye was added to each PCR product and the mixture was loaded onto a 10% TBE-PAGE gel (Thermo Fisher Scientific). Then, the cDNA was isolated from the ~150 bp gel band. After the size selection, the quality and quantity of individual libraries were assessed using Agilent RNA high sensitivity screen tape kits and Bioanalyzer 2200 TapeStation system. The individual libraries were pooled at equimolar ratio and sequenced as mentioned above.

### Bioinformatics Analyses and Statistics

In our related study on AIC (8), we profiled the expression of selected cell-specific genes (macrophages, dendritic cells, T and B cells and endothelial cells) and genes related to cytokines and chemokines. In the present study, we reanalysed the same mRNA transcriptome data presented in the aforementioned paper and integrated it with the newly generated small RNA-Seq dataset. In particular, i) we filtered out top thirty equally abundant macrophage-related genes (with |Log_2_FC)| < 0.005) and miRNAs (with |Log_2_FC| < 1) in AIC and AKC, ii) determined differentially expressed genes and miRNAs between AIC and AKC, and iii) analysed the association between mRNAs and miRNAs (i.e., correlation analysis between miRNA and their target gene).

### mRNA-Seq and Small RNA-Seq Data Analyses

All bioinformatic analyses of mRNA-Seq data were performed as previously described by Zhang, Kopp (27). Briefly, raw data were converted to fastq format with bcl2fastq2 (v2.17, Illumina), followed by adapter trimming using Cutadapt (28) and filtering low-quality reads with fastq_quality_filter “-q 20 -p 80”. The clean reads were then aligned against ICSASG_v2 assembly of Atlantic salmon genome using STAR (29) with the following parameters: “–outSAMtype BAM SortedByCoordinate –quantMode TranscriptomeSAM GeneCounts”. Read counts were extracted from the alignment file ReadsPerGene.out.tab, the fourth column of which refers to the read counts from the “2^nd^ read strand”. Raw read counts of the 12 samples were merged to create a matrix, which was used for the differential expression analysis. For small RNA-Seq analysis, the quality of raw reads was assessed using FastQC (30), followed by adapter trimming using Cutadapt and removal of low quality reads using FASTX-Toolkit as described above. Clean reads were aligned to Atlantic salmon genome ICSASG_v2 using miRDeep2 package (31) command mapper.pl with parameters: “-e -d -h -i -j -l 18 -m -n -o 16”, and the counts were generated using command quantifier.pl with salmon hairpin and mature miRNAs from miRbase (32) as input. Details of raw, clean and mapped reads are provided in **Supplementary Table 1**. The filtered miRNAs were employed to reveal the differential expression between the genes in AIC to AKC. DESeq2 (33) was employed to determine differentially expressed mRNAs and miRNAs, after clarifying the dispersion estimates and minus over average expression (**Supplementary Figure 1**). An absolute fold change (FC) ≥ 2 and Benjamini-Hochberg adjusted p value < 0.05 were considered for the analyses. The R packages ggplot2 (34) and pheatmap (35) were employed for data visualization. Enrichment of KEGG pathways and gene ontology based on the up or downregulated genes were performed using clusterProfiler (36) as described previously (37). Here, an absolute FC > 4 was considered for the analyses. The R packages ggplot2 (34) and ggraph (38) were used to generate the graphs.

### *In Silico* Analysis to Determine the Target Genes of miRNAs

To study the correlation between DE miRNAs and their target transcripts found in the DEGs, we used two target prediction algorithms: miRanda “-sc 140 -en -20 -scale 4 -strict -go -4 -ge -9 -quiet” (39) and RNAhybrid “-b 1 -c -m 5000 -u 1 -v 1 -e -20 -p 0.05 -s 3utr_human -q” (40). Only genes that were identified by both algorithms were selected as potential targets. We focused on miRNAs and their target pairs that were inversely correlated since miRNAs are negative regulators of gene expression (41). Using Pearson’s correlation analysis, negative correlations between DE miRNA and their target mRNAs among DEGs were determined according to the following three criteria: (1) normality of the data and homoscedasticity of residuals, (2) correlation coefficient r < 0 and (3) p-value < 0.05. Here we considered two types of negative correlation pairs: (1) upregulated DE miRNAs and downregulated target genes among DEGs, (2) downregulated DE miRNAs and upregulated target genes among DEGs.

## Results

### Macrophage-Like Cells With Phagocytic Activity

**Figure 1:**
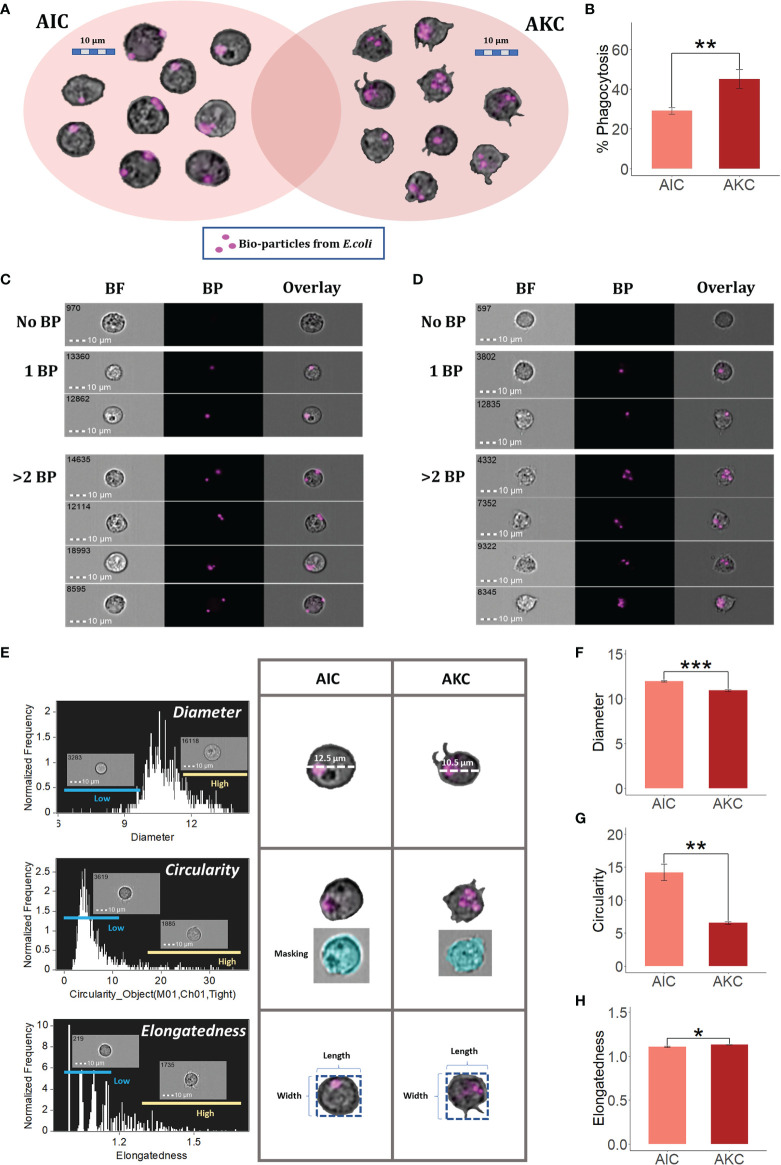
Phagocytes in the adherent cells from the intestine and head kidney of Atlantic salmon. **(A)** Macrophage-like cells that exhibited phagocytic activity. **(B)** Phagocytic activities of macrophage-like cells in AIC and AKC. Representative cell images show macrophage-like cells with no bioparticles (BP), 1BP and >2BP in AIC **(C)** and AKC **(D)**. Workflow of measuring diameter, circularity and elongatedness of phagocytes in AIC and AKC **(E)**. Diameter **(F)**, circularity **(G)** and elongatedness **(H)** of phagocytes from AIC and AKC. Bar plots indicate the mean ± SD (*n = 6*). Significant differences are indicated using asterisks: *p < 0.05, **p < 0.01, ***p < 0.001. All cell images were captured with 40 × objectives. Scale bar = 10 µm. AIC, adherent cells from the distal intestine; AKC, adherent cells from the head kidney; BF, brightfield; 1BP and >2BP, 1-2 internalized particles.

Based on the protocol described in our previous studies (7, 8), we were able to identify the area of macrophage-like cells in the cell population plot (bright-field area vs side scatter intensity). Gates of macrophage-like cells in the plots of AIC and AKC populations were set based on the cell size and granularity (**Supplementary Figures 2A, B**). AKC had more stretched cells while AIC had relatively circular cells. Furthermore, we employed PI staining to show the shapes of nuclei of macrophage-like cells in AIC (**Supplementary Figure 2C**). We also measured the phagocytic activity of the macrophage-like cells in the above-mentioned gates using the imaging flow cytometry. **Figures 1A, B** indicate the phagocytic activities of the macrophage-like cells in both AIC and AKC populations. In addition, we have generated representative cell images of such phagocytes that have engulfed different numbers of *Escherichia coli* bioparticles (**Figures 1C, D**). As for the cell shapes, we employed features and mask in the IDEAS software to understand the morphology of the phagocytes in AIC and AKC (**Figure 1E**). Diameter and circularity of the phagocytes in AIC were significantly higher than those in AKC (**Figures 1F, G**). However, the phagocytes in AKC were more stretched or elongated compared to those in AIC (**Figure 1H**).

### Macrophage-Related mRNA and miRNA Genes

To reveal the presence of plausible macrophage-like cells, in the present study, we employed the mRNA transcriptomes of the AIC and AKC that were used in our previous study (8). In addition, miRNA transcriptomes of the same pool of AIC and AKC (8) were targeted to obtain a better understanding of how miRNAs regulate their target macrophage-related genes. It should be noted that the whole adherent cell pools in the intestine and head kidney were employed for the transcriptome study. We used only a subset of this pool to study the phagocytosis of macrophage-like cell population.

**Figure 2:**
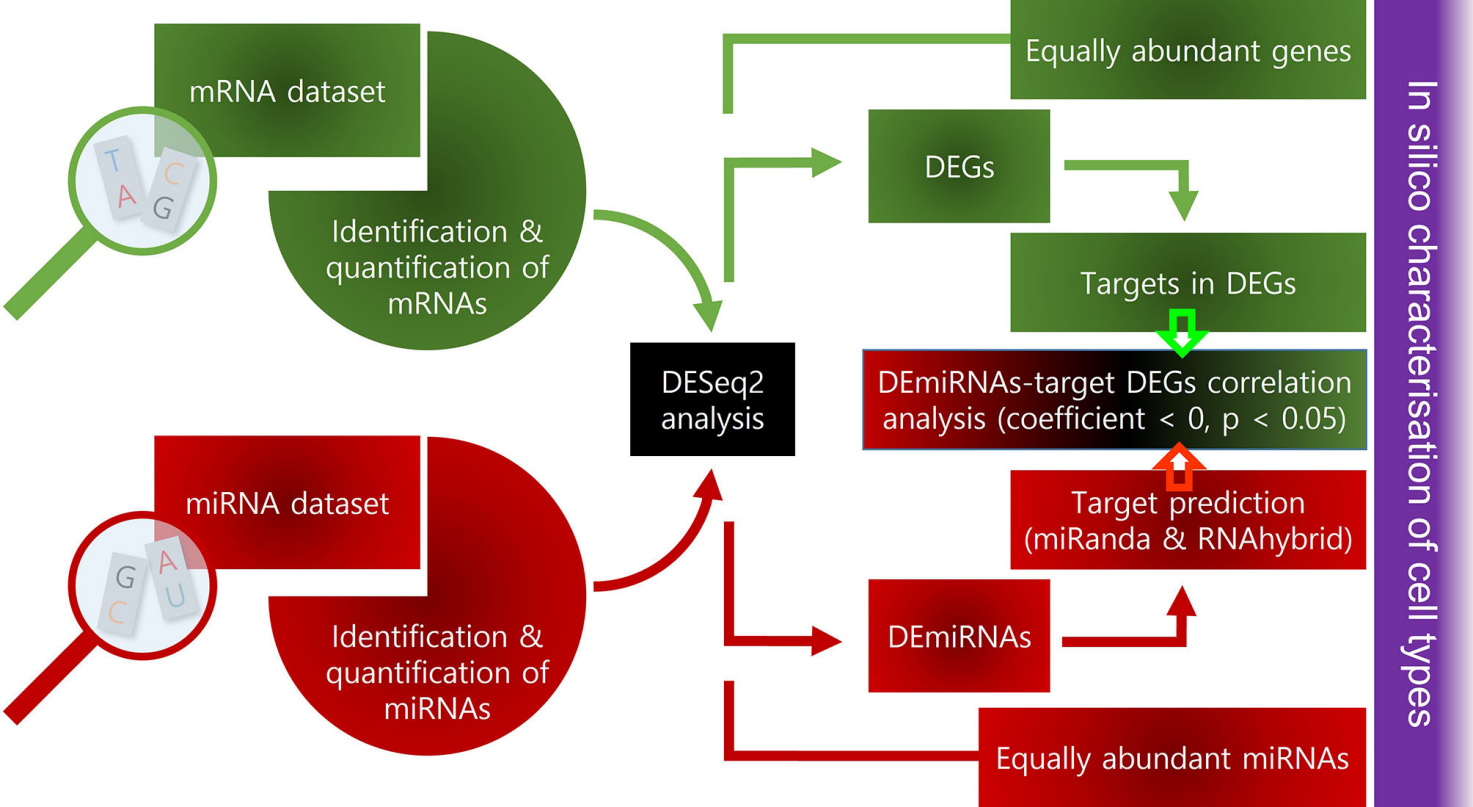
Workflow of the bioinformatic analysis of mRNA and small RNA sequencing datasets.

**Figure 3:**
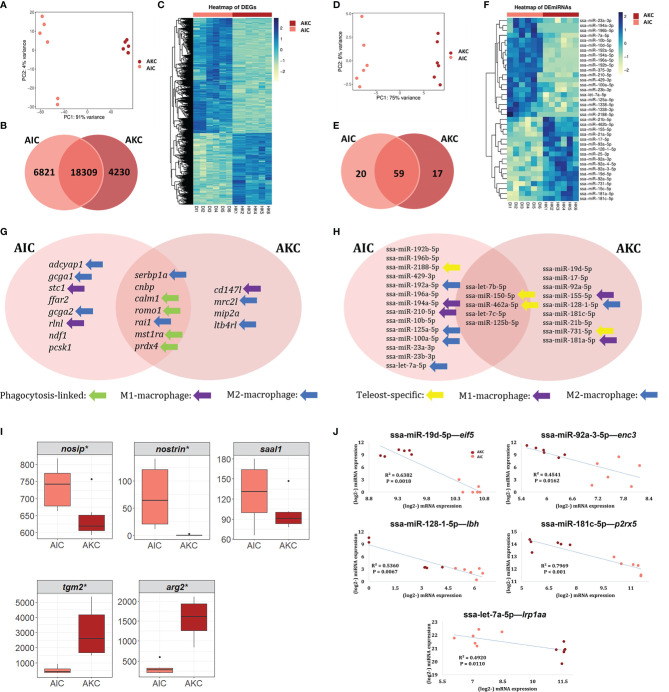
Markers of macrophage-like cells in the mRNA or miRNA transcriptomes of the adherent cells from the intestine and head kidney of Atlantic salmon. Differences in the mRNA and miRNA transcripts **(A, C, D, F)**. Venn diagram showing the number of common and unique genes in mRNA **(B)** or miRNA **(E)** transcriptomes. Macrophage-related mRNAs and miRNAs among top 20 genes in AIC **(G)** and AKC **(H)** populations. Employing the normalized read counts from DESeq2 analyses, the expression levels of the potential gene markers of fish M1- (**I**, upper) and M2-macrophages (**I**, lower) were compared to understand the phenotypes in AIC and AKC. Statistically significant differences (* p value < 0.05) are indicated using asterisks. Boxplots show the median, minimum and maximum values in the data (n = 6). **(J)** Negative correlations between the miRNA and mRNA. AIC, adherent cells from the distal intestine; AKC, adherent cells from the head kidney; *nosip*, nitric oxide synthase interacting protein; *nostrin*, nitric oxide synthase trafficking; *saal1*, serum amyloid A-like 1; tgm2, transglutaminase 2, like; *arg2*, arginase 2; *eif5*, eukaryotic translation factor 5-like; *enc3*, ectodermal-neural cortex 3; *lbh*, protein LBH-like; *p2rx5*, P2X purinoceptor 5-like; *lrp1aa*, low-density lipoprotein receptor-related protein 1-like.

The overall workflow of the mRNA-Seq and small RNA-Seq data analysis is shown in **Figure 2**. The principal component analysis plots of mRNA (**Figure 3A**) and miRNA (**Figure 3D**) sequence data show two clear clusters corresponding to AIC and AKC. However, there were many common mRNA and miRNAs in AIC and AKC. While the number of common mRNA was 18309 (**Figure 3B**), the corresponding number for the miRNA (**Figure 3E**) was 59. The macrophage-related mRNAs and miRNAs among top 30 equally abundant mRNAs or miRNAs are shown in **Figures 3G**, **H**, respectively. Of the genes that had similar normalised read counts in AIC and AKC (**Supplementary Table 2**), *serbp1a*, *ran1* and *cnbp* were the most abundant (with high base mean) genes. Furthermore, six genes, namely *mst1ra*, *romo1*, *prdx4, calm1*, *bcam* and *ceacam18* were equally abundant in AIC and AKC. Among the similarly expressed miRNAs in the two types of adherent cells (**Supplementary Table 3**), ssa-let-7b-5p and ssa-miR-150-5p were the most abundant small RNAs.

### Characteristic Genes of the Adherent Intestinal and Head Kidney Cells

As for the differentially expressed mRNAs, in total, 11051 DEGs were identified in AIC compared to AKC; 6821 up and 4230 downregulated DEGs (**Figures 3B, C** and **Supplementary Table 4**). In addition, 37 DE miRNAs were identified in AIC compared to AKC; 20 up and 17 downregulated DE miRNAs (**Figures 3E, F** and **Supplementary Table 5**). Several macrophage-related genes were found among the top 20 up and downregulated mRNAs or miRNAs in AIC (**Figure 3G**) and AKC populations (**Figure 3H**).

Among the upregulated DEGs (**Figure 3G**), there were two highly regulated genes (log_2_FC ≥ 13.5) in AIC compared to AKC; *adcyap1* and *epcam*. In addition, *gcga1*, *stc1*, *segn*, *tm4sf4*, *t4s1*, *ffar2*, *gcga2*, *rlnl* and *scg3* that were detected as upregulated DEGs in AIC also had high fold changes (> 12). As for the downregulated DEGs (**Figure 3G**), there were two genes that were highly downregulated in AIC compared to AKC; *acsl3l* and *p2ry12l* (log_2_FC ≤ -8.9). Furthermore, some other genes were also downregulated; *cd147l*, *mrc2l*, *mip2a*, and *ltb4rl* (log_2_FC ≤ -6.7).

The three most upregulated miRNAs (log_2_FC ≥ 12) in AIC compared to AKC were ssa-miR-192b-5p, ssa-miR-196b-5p and ssa-miR-2188-5p. The 20 DE miRNAs that had log_2_FC in the range 1-13 included ssa-miR-429-3p, ssa-miR-192a-5p, ssa-miR-196a-5p, ssa-miR-194a-5p, ssa-miR-210-5p, ssa-miR-10b-5p, ssa-miR-125a-5p, ssa-miR-100a-5p, ssa-miR-23a-3p, ssa-miR-23b-3p and ssa-let-7a-5p (**Figure 3H**). The three most downregulated miRNAs (log_2_FC ≤ -4.3) in AIC compared to AKC were ssa-miR-19d-5p, ssa-miR-17-5p and ssa-miR-92a-5p. The 17 downregulated DE miRNAs that had log_2_FC in the range between -1 and -9.7 included ssa-miR-155-5p, ssa-miR-128-1-5p, ssa-miR-181c-5p, ssa-miR-21b-5p, ssa-miR-731-5p and ssa-miR-181a-5p (**Figure 3H**).

### Macrophage Heterogeneity in the Adherent Intestinal and Head Kidney Cells

This is our first exploration to understand the heterogeneity of the macrophages in AIC and AKC. Five candidate markers for carp M1- and M2-macrophages that were identified by Wentzel, Petit (25) were detected in the transcriptome of AIC and AKC (**Figure 3I**). AIC had higher expression of M1-macrophage markers (*nosip*, *nostrin* and *saal1*) while AKC had higher expression of M2-macrophage markers (*tgm2* and *arg2*).

### Integrative Analysis of DE miRNA and Their Target Genes in DEGs

Integrative analysis of DE miRNAs and their target genes among DEGs revealed 15 negative correlation pairs (**Table 1**). Of them, 5 pairs were significantly correlated (p < 0.05; 4 pairs: downregulated DE miRNA—upregulated target gene, and 1 pair: upregulated DE miRNA—downregulated target gene); ssa-miR-19d-5p—*eif5*, ssa-miR-92a-3-5p—*enc3*, ssa-miR-128-1-5p—*lbh*, ssa-miR-181c-5p—*p2rx5* and saa-let-7a-5p—*lrplaa* (**Figure 3J**).

**Table 1:** Negative correlations between differentially expressed miRNAs and their potential target mRNAs among DEGs.

MicroRNA	Target gene	Symbol	Description	Correlation coefficient (r)	P value
AIC—downregulated miRNA & upregulated target gene					
**ssa-miR-181c-5p**	LOC106612812	***p2rx5***	**P2X purinoceptor 5-like**	-0.8927	<0.001
**ssa-miR-19d-5p**	LOC106607644	***eif5***	**Eukaryotic translation initiation factor 5-like**	-0.7989	0.0018
**ssa-miR-128-1-5p**	LOC106571118	***lbh***	**Protein LBH-like**	-0.7321	0.0067
**ssa-miR-92a-3-5p**	LOC106560599	***enc3***	**Ectodermal-neural cortex 3**	-0.6739	0.0162
ssa-miR-92a-3-5p	LOC106589523	*eef2k*	Eukaryotic elongation factor 2 kinase-like	-0.6015	0.0385
ssa-miR-128-1-5p	LOC106574722	*cu051*	Small integral membrane protein 11-like	-0.5774	0.0492
ssa-miR-92a-4-5p	LOC106611553	*qdpra*	Dihydropteridine reductase-like	-0.5728	0.0515
ssa-miR-93a-5p	LOC106576270	*spic*	Transcription factor Spi-C-like	-0.5566	0.0601
ssa-miR-92a-3-5p	LOC106603902	*fam53c*	Family with sequence similarity 53 member C	-0.5525	0.0624
ssa-miR-92a-3-5p	LOC106612663	*fam83b*	Protein FAM83B-like	-0.5459	0.0663
ssa-miR-92a-4-5p	LOC106562770	*call4*	Calmodulin-like protein 4	-0.4746	0.119
ssa-miR-92a-3-5p	LOC106607421	*pbx4*	Pre-B-cell leukemia transcription factor 1-like	-0.4538	0.1384
ssa-miR-92a-4-5p	LOC106601006	*fbxl20*	F-box/LRR-repeat protein 20	-0.3896	0.2105
ssa-miR-92a-4-5p	LOC106572667	*lmbr1l*	Limb region 1 homolog-like protein	-0.3423	0.2761
AIC—upregulated miRNA & downregulated target gene					
**ssa-let-7a-5p**	LOC106565466	***lrp1aa***	**Low-density lipoprotein receptor-related protein 1-like**	-0.7014	0.0110

Significant negative correlations between microRNAs and their target genes are shown in bold.

### Predicted Functional Characteristics of Adherent Intestinal and Head Kidney Cells

**Figure 4:**
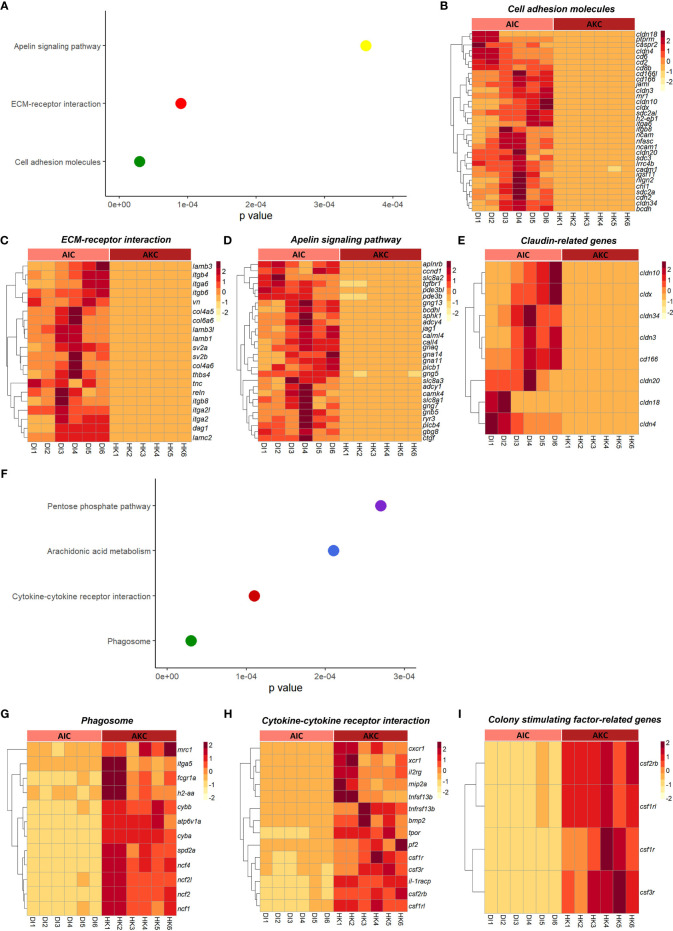
Enriched functions associated with the adherent cells from the intestine and head kidney of Atlantic salmon. **(A)** Enriched KEGG pathways based on the upregulated genes. Heatmaps showing the genes associated with macrophage-related pathways like **(B)** cell adhesion molecules, **(C)** ECM-receptor interaction **(D)** apelin signaling pathway and **(E)** claudin-related genes. **(F)** Enriched KEGG pathways based on the downregulated genes. Heatmaps show the differential expression of genes associated with macrophage-related pathways like **(G)** phagosome, **(H)** cytokine-cytokine receptor interaction and **(I)** colony stimulating factor-related genes. AIC, adherent cells from the distal intestine; AKC, adherent cells from the head kidney.

**Figure 5:**
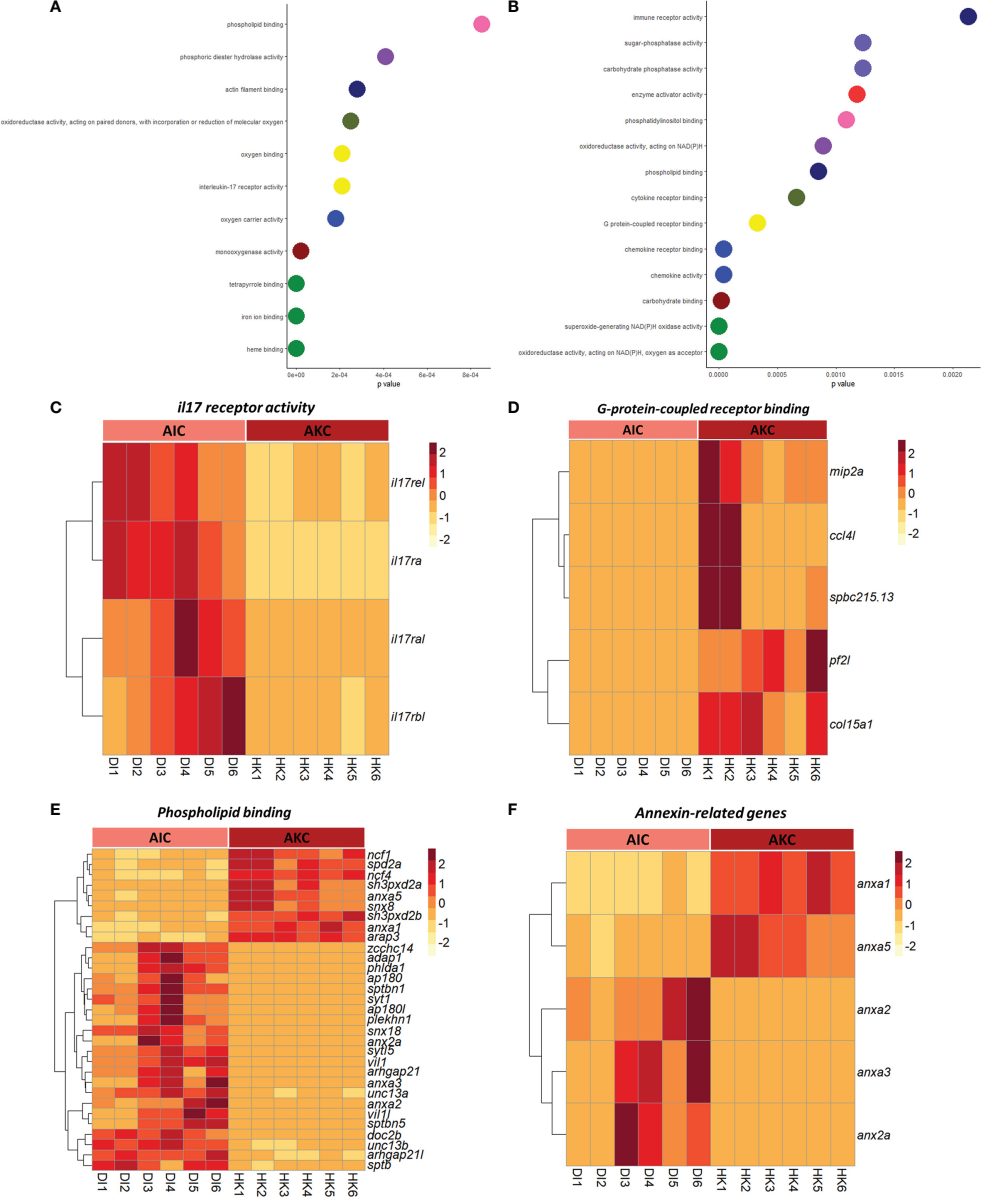
Enriched GO terms associated with the adherent cells from the intestine and head kidney of Atlantic salmon. Enriched GO terms based on the upregulated **(A)** or downregulated **(B)** genes. Heatmaps showing the genes associated with macrophage-related GO terms like **(C)** il17 receptor activity **(D)** G-protein-coupled receptor binding **(E)** phospholipid binding and **(F)** annexin-related genes based on both up or downregulated genes. AIC, adherent cells from the distal intestine; AKC, adherent cells from the head kidney.

The enriched pathways by considering the upregulated genes in AIC compared to AKC included cell adhesion molecules, extracellular matrix-receptor interaction, and apelin signaling pathway (**Figures 4A**–**D**). As for the enriched pathways based on the downregulated genes in AIC, we detected pathways like phagosome, and cytokine-cytokine receptor interaction (**Figures 4F**–**H**). Among the genes involved in the abovementioned pathways, genes linked to claudin were highly expressed in AIC compared to AKC (**Figure 4E**), while colony stimulation factor-related genes were downregulated in AIC (**Figure 4I**). GO term analysis employing the upregulated genes in AIC revealed several enriched terms; they belonged to many binding proteins such as phospholipid binding and interleukin-17 receptor activity (**Figures 5A, C, E**). Downregulated genes in AIC-linked GO terms included enzyme activities such as chemokine receptor binding, cytokine receptor binding, G protein-coupled receptor binding and phospholipid binding (**Figures 5B, D, E**). Several annexin-related genes were found to be contributing to the aforementioned pathways; while *anxa1* and *anxa5* were downregulated, *anxa2*, *anxa2a*, and *anxa3* were upregulated in AIC compared to AKC (**Figure 5F**). In addition, we found that several genes that are known to be associated with intestinal muscularis macrophages were upregulated in AIC; purine-related genes (*p2rx5*, *5ntc*, *p2rx4*, *purg*, and *p2rx1*), nicotinic-related gene (*nmnat2*) and acetylcholine-related genes (*chrna5*, *chrm2*, *slc18a3a*, and *chrna7*) (**Supplementary Figure 3**).

## Discussion

Teleost fish intestinal immune cell types are not characterized thoroughly because of the scarcity of appropriate antibodies and complexity of techniques to be adopted for such discovery. We have been characterising Atlantic salmon intestinal cells employing novel approaches. Our strategy has been to exploit the ability of the intestinal cells to adhere to surfaces, specifically, the adherent cells from the distal intestine (AIC) and distinguish these by gene expression. Adopting this approach, we previously found that AIC express several macrophage-specific genes (8). In the present study, we further examined macrophage-like cells in the AIC population for phagocytic activity and cell shape. In addition, we identified equally abundant genes in both AIC and adherent cells from head kidney (AKC or macrophage-like reference cells) as well as differentially expressed (DE) mRNAs and miRNAs in AIC compared to AKC. Analysis of DE miRNAs and DE mRNAs revealed several enriched pathways and GO terms. In addition, correlation analysis of these differentially expressed genes revealed the negatively correlated pairs in the intestinal macrophages of salmon.

### Macrophage-Like Cells in AIC

Adherent HK cells from Atlantic salmon are known to contain macrophages originating from monocytes (42). The origin of macrophage-like cells in AIC is yet to be revealed; some are likely to be resident while others could be freshly recruited for a particular function. In mammals, intestinal macrophages interact with commensal gut microbiota and antigens from both food and pathogenic microbes (43). Depending on the required tolerance or activation of the immune system, macrophages get polarized into M1 and M2 phenotypes with distinct morphology (44). Our study showed heterogeneity of macrophages in AIC and AKC; macrophage populations comprised both M1- and M2-like phenotypes. For example, the genes *serbp1a* (plasminogen activator inhibitor 1 RNA-binding protein-like) and *cnbp* (CCHC-type zinc finger, nucleic acid binding protein) were equally abundant in both, AIC and AKC. The former gene is known to recruit macrophages and polarize them into M2 forms (45) while the latter is associated with the cytosol of macrophages and is a key regulator of transcription of c-Rel–dependent IL-12β gene and Th1 immunity (46). The gene encoding adenylate cyclase activating polypeptide 1, *adcyap1*, was upregulated in AIC. This cytoprotective peptide modulates murine macrophage polarization during chronic inflammation—increased M2 polarization but reduced M1 polarization by interfering with JNK/STAT3 signalling pathway (47). Two other genes were downregulated in AIC, namely *mrc2l* (C-type mannose receptor 2-like) and *ltb4rl* (leukotriene B4 receptor 1-like). The former is known to be expressed by mouse M2-like macrophages (48), and *ltb4rl* is expressed by mouse macrophages favouring M2 polarization (49). On the contrary, two other genes upregulated in AIC, namely *stc1* (stanniocalcin 1) and *rlnl* (relaxin-like), point to the existence of M1 macrophages. A recent study reported that lipopolysaccharide (LPS)/IFNγ-induced M1 human macrophages had higher expression levels of STC1 compared to phorbol myristate acetate-induced M0- and IL-4/IL-13-induced M2-macrophages (50). Furthermore, *rlnl* (relaxin-like protein), is known to significantly increase pro-inflammatory cytokine IL-6 in human M1 macrophages (51). Murine and human M1 macrophages that express *cd147* induced Th17 differentiation (52) and this gene was downregulated in AIC. In our study, GO terms for upregulated genes in AIC included *il17* receptor activity because of the induction of several *il17* receptor-related genes (*il17rel*, *il17ra*, *il17ral*, and *il17rbl*), suggesting that AIC could have stronger Th17 responses than AKC. A study on IL-17 knockout mice (53) showed that IL-17 is associated with M2-like macrophages to protect against DSS-induced colitis. The head kidney and intestine cells of Atlantic salmon had similar expression of *mst1ra*. This gene, encoding macrophage-stimulating protein receptor, is expressed on mammalian peritoneal macrophages (54) and regulates their biological functions, including phagocytosis and M1-M2 macrophage balance (55).

In the present study, adherent cells in AIC and AKC had different morphologies; the cells in AIC were larger and more circular than those of AKC. On the contrary, the cells in AKC were more stretched compared to those of AIC. Mammalian studies have reported that following migration from the bloodstream into the tissues, bone marrow-derived monocytes enlarge and differentiate into resident macrophages by increasing their lysosomal and hydrolytic enzymes and mitochondrial number and size (56). However, it should also be noted that replenishment of tissue-resident macrophages can take place locally and independent of macrophages derived from circulating monocytes (57, 58). In a study on mice (59), tissue-resident macrophages that did not originate from bone marrow were larger in size and more circular. A study by Leavy (44) found that macrophage elongation is a characteristic of M2 macrophages that have higher expression levels of arginase 1 and lower expression of nitric oxide synthase. In our study, AKC had higher expression of *arg2* (arginase 1) and *tgm2* (transglutaminase 2) and lower expression of two nitric oxide synthase-related genes; *nosip* (nitric oxide synthase-interacting protein), *nostrin* (nitric oxide synthase trafficking) and *saal1* (serum amyloid A like 1) compared to AIC. Human studies revealed the increase in expression of transglutaminases 2, known as a marker of M2-macrophages, during the monocyte-to-macrophage differentiation (60, 61). Our previous studies on teleost common carp macrophages identified potential markers for M1-macrophages (*il1b*, *nos2b* and *saa*) and M2-macrophages (*timp2b*, *tgm2b* and *arg2*) based on LPS and cAMP stimulation, respectively, with associated differences in nitric oxide production and arginase activity (25). Here, we noted differential expression of the genes *nosip* and *nostrin*, both involved in modulating nitric oxide synthesis (62, 63). Our data indicated that phenotypical changes in macrophages could be closely related to the cell shapes.

There are several studies in mammals that describe the epigenetic control and shaping of macrophage phenotypes. A study on mice (64) showed that by inhibiting the histone deacetylase activity, macrophage differentiation (M2 phenotype) and morphology (towards an elongated shape) can be altered. Hence, miRNAs, the epigenetic modulators, also play a key role in shaping the macrophage shapes and phenotypes. We did not study histone deactylase activity but investigated miRNA levels in the two cell types, because it is known that histone deacetylase activity can affect gene expression (miRNA and mRNA) (65). The miRNAs that were equally or differentially abundant in AIC and AKC included ssa-let-7b-5p, ssa-miR-125b-5p, ssa-let-7c-5p and miR-23a-3p. In mammalian macrophages, let-7b (66), let-7c-5p (67) miR-125b (68) and miR-23a-3p (69) have been associated with macrophage polarization. Some of the upregulated miRNAs in AIC have been found to be associated with M2 polarization. One of them was ssa-miR-100a-5p, and miR-100 can induce the M2-polarization of macrophages (70) and high expression of let-7a upregulated the anti-inflammatory factors and promoted the switch from M1 to M2 phenotype (71). ssa-miR-125a-5p was among the top upregulated miRNAs in AIC and high expression of miR-125 has been reported in mice M2-macrophages (72). In addition, ssa-miR-192a-5p was upregulated in AIC, as observed for bone marrow-derived macrophages of mice, wherein the expression of miR-192 promoted M2-macrophage differentiation *in vitro* (73). In salmon, ssa-miR-462a-5p was expressed during head kidney monocyte-to-macrophage differentiation (15). Yet another miRNA upregulated in AIC is ssa-let-7a-5p and its target gene is *lrp1aa*. Mueller, Zhu (74) found that low-density lipoprotein receptor-related protein (*lrp*) controls the expression of C-C chemokine receptor type 7 (*ccr7*, a macrophage polarization marker) in mice macrophages. LRP1-mediated signalling helps in the resolution of active inflammatory response and promotes the conversion to anti-inflammatory M2 functional phenotype (75). In addition, LDL receptor-related protein-1 is known to regulate miR155 (76). It should be noted that ssa-miR-155-5p was downregulated in AIC. As of the upregulated genes in AIC, miR-196b-5p (77), and miR-196 (78) were associated with macrophage activation. Eukaryotic translation initiation factor-5 (*eif5*) is the target gene of ssa-miR-19d-5p and it was downregulated in AIC, and *eif5* is known to modulate mitochondrial respiration and indirectly affect alternative (M2) macrophage activation in humans (79). Hence, we believe that macrophage polarization is regulated by miRNA.

As for the G-protein-coupled receptors on intestinal macrophages, they are closely linked to neurotransmitters (80, 81). In our study, although the expression of G-protein-coupled receptors were lower in AIC, we observed higher expression of two neurotransmitter-related genes, *ndf1* and *pxsk1*. It should be noted that intestinal muscular macrophages that are located near enteric neurons have many neurotransmitter receptors for example purinoceptors, nicotinic and acetylcholine receptors (82). A recent study revealed that lamina propria as well as muscularis-associated macrophages were significantly reduced (50 and 95%, respectively) in *irf8*-deficient zebrafish (83). Although *irf8* was downregulated in AIC, certain other genes linked to muscularis macrophages were upregulated in AIC; purine-related genes (*p2rx5*, *5ntc*, *p2rx4*, *purg*, and *p2rx1*), nicotinic-related gene (*nmnat2*) and acetylcholine-related genes (*chrna5*, *chrm2*, *slc18a3a*, and *chrna7*). Taken together, the intestinal macrophage pool that we isolated presumably contains muscularis macrophages and have phagocytic ability as stated about the tissue-resident macrophages by De Schepper, Stakenborg (84).

### Other Cell Types Required for Macrophage Activation

Several genes that were upregulated in AIC can be linked to structural cells like epithelial cells and endothelial cells. Two cell adhesion-related genes, namely *bcam* and *ceacam18* were expressed in both, AIC and AKC. In mice, basal cell adhesion molecule (*bcam*) plays crucial roles in facilitating the accumulation of monocytes and macrophages (85). Our functional analysis revealed that enriched pathways based on the upregulated genes in AIC included pathways of cell adhesion molecules, and pathways of extracellular matrix-receptor interaction. An *in vitro* study on human cell line (86) reported that during monocyte-macrophage differentiation, macrophages produce proteoglycans that can control cell adhesion and formation of extracellular matrix. The top DEGs in AIC included genes associated with endothelial (*segn* and *scg3*) (87, 88) and epithelial cells (*epcaml*, *tm4sf4* and *t4s1*) (89). Chi and Melendez (90) reported that an interplay between monocytes and endothelial cells is mediated by intercellular or vascular cell adhesion molecules. Furthermore, Prieto, Eklund (91) reported that the maturation of monocytes to macrophages is regulated by cell adhesion molecules.

In conclusion, the present flow cytometric and transcriptomic studies have provided new cellular and molecular insights on the intestinal adherent cells of Atlantic salmon. Flow cytometry data revealed the differences between the morphologies of AIC and AKC. The transcriptome data provided information of specific miRNA and mRNA, and this knowledge was used to indicate the existence of both M1 and M2 macrophages in the adherent intestinal cells of salmon. Taken together, we have revealed the functional characteristics of the macrophages isolated from the two organs; in terms of their phagocytic activity, polarizations, and interaction with structural cells like epithelial cells and endothelial cells. However, the comparison that has been done (AIC vs AKC, without stimuli) is not based on a pure macrophage population and other cell types can be found in the adherent cell populations. For example, non-professional phagocytes like epithelial cells, endothelial cells and fibroblasts are adherent cell types (92–94) with high phagocytic activity (95). Furthermore, the cell types other than macrophage-like cells will be very different in head kidney and distal intestine and may account for some of the DEGs. It should also be noted that although we tried to classify M1 and M2 macrophages based on transcriptome data, there could be a wide macrophage diversity spectrum, and possibly not just a switch between the two complex phenotypes. Nevertheless, there occurs a switch between M1 and M2 depending on health and disease conditions (96, 97). Ideally, other tools like single-cell RNA sequencing or RNA *in situ* hybridisation can provide a better understanding of intestinal macrophage populations and functions. The adherent cell population reported here should be further studied to understand their contribution to intestinal immunity in Atlantic salmon.

## Data Availability Statement

mRNA-Seq and small RNA-Seq data can be found in Gene Expression Omnibus (GEO, NCBI) under the accession numbers GSE154142 and GSE154147, respectively. All in-house scripts can be obtained from the authors on request.

## Ethics Statement

The present study was approved by the National Animal Research Authority in Norway (Mattilsynet; FOTS ID 10050) and all the protocols were according to its guidelines.

## Author Contributions

YP, GW, JF, and VK conceived and designed the study. YP and QZ performed the experiment, RNA sequencing and data analysis. YP wrote the first draft of this manuscript. YP, QZ, GW, JF, and VK read and revised the manuscript. All authors contributed to the article and approved the submitted version.

## Funding

This study was partially supported by INFISH project (272004) funded by the Regionale Forskningsfond Nord-Norge. YP was a recipient of the Korean Government Scholarship—National Institute for International Education, South Korea.

## Conflict of Interest

The authors declare that the research was conducted in the absence of any commercial or financial relationships that could be construed as a potential conflict of interest.

## Publisher’s Note

All claims expressed in this article are solely those of the authors and do not necessarily represent those of their affiliated organizations, or those of the publisher, the editors and the reviewers. Any product that may be evaluated in this article, or claim that may be made by its manufacturer, is not guaranteed or endorsed by the publisher.

## Supplementary Material

Supplementary Figure 1 Dispersion estimates and minus over average expression of the mRNA-Seq **(A, B)** and small RNA-seq **(C, D)** dataset, respectively.

Supplementary Figure 2 Identification of macrophage-like cells in the AIC and AKC populations using imaging flow cytometry. Macrophage-like cells were identified based on the morphologies of cells and shapes of the nuclei. The areas of macrophage-like cells in AIC **(A)** and AKC **(B)** were gated in a bright field (BF) area (cell size) vs. side scatter (SSC) intensity (cell granularity) plot. Proportions of the macrophage-like cells among the whole adherent cell pool is indicated in the figure. Representative images from each gate are shown; **(C)** Propidium iodide (PI) staining revealed the shapes of the nuclei of intestinal macrophage-like cells in AIC **(C)**. All cell images were captured with 40 × objective. Scale bar = 10 μm.

Supplementary Figure 3 Heatmap showing muscularis macrophage-related genes that were upregulated in adherent cells from the distal intestine of Atlantic salmon. AIC: adherent cells from the distal intestine; AKC: adherent cells from the head kidney; *p2rx5*: P2X purinoceptor 5-like; *5ntc*: cytosolic purine 5-nucleotidase; *p2rx4*: P2X purinoceptor 4-like; *purg*: purine-rich element-binding protein gamma pseudogene; *p2rx1*: P2X purinoceptor 1-like; *nmnat2*: nicotinamide/nicotinic acid mononucleotide adenylyltransferase 2; *chrna5*: neuronal acetylcholine receptor subunit alpha-5-like; *chrm2*: muscarinic acetylcholine receptor M2-like; *slc18a3a*: probable vesicular acetylcholine transporter-A; *chrna7*: neuronal acetylcholine receptor subunit alpha-7-like.
