# Supplementary figures and images for: Macrophage Heterogeneity in the Intestinal Cells of Salmon: Hints From Transcriptomic and Imaging Data
Source: Front Immunol. 2021 Dec 23;12:798156. doi: 10.3389/fimmu.2021.798156 (PMC8733388; doi:10.3389/fimmu.2021.798156)

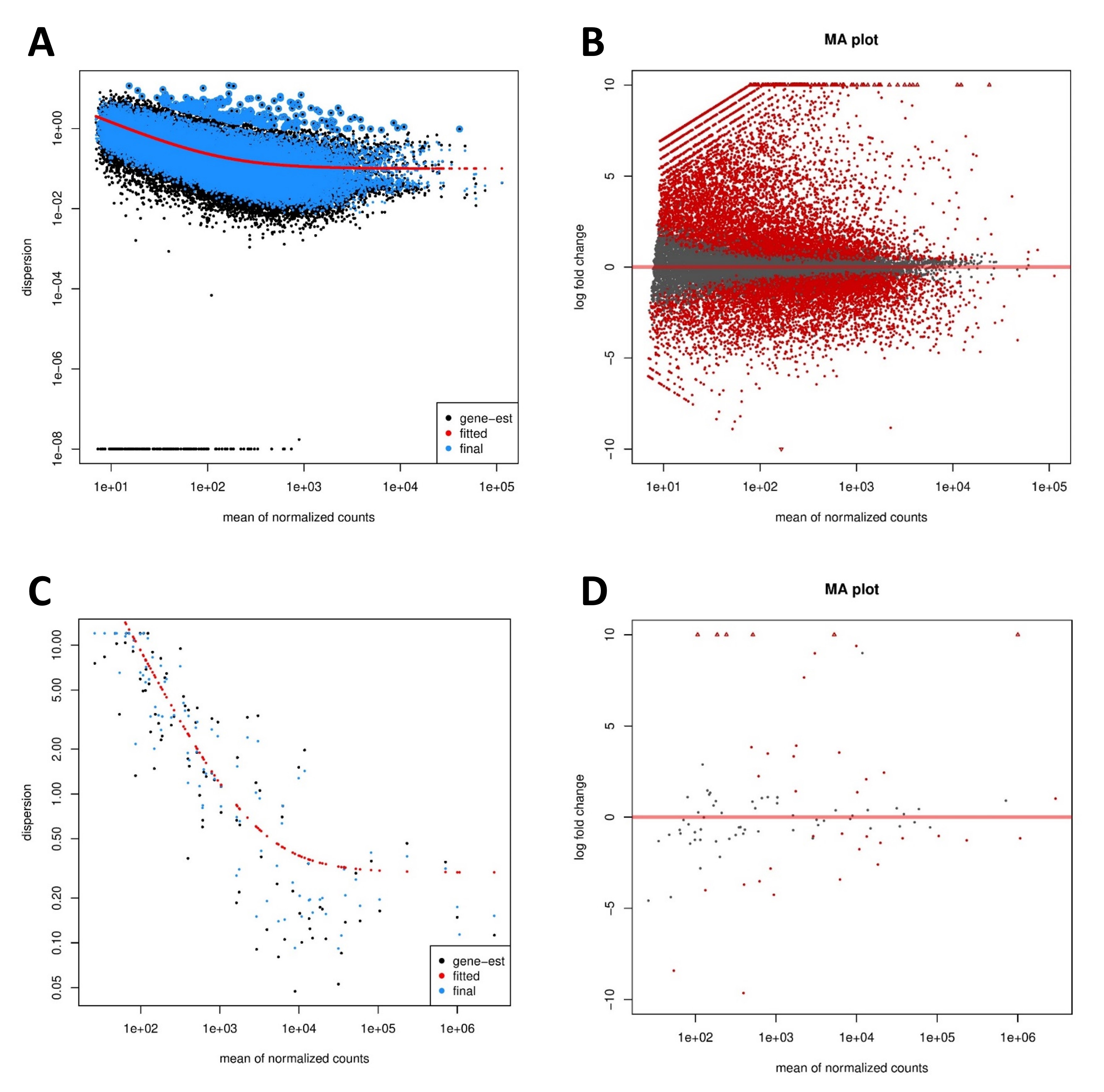

Supplement: Supplementary Figure 1 — Dispersion estimates and minus over average expression of the mRNA-Seq (A, B) and small RNA-seq (C, D) dataset, respectively. [file Image_1.jpeg]

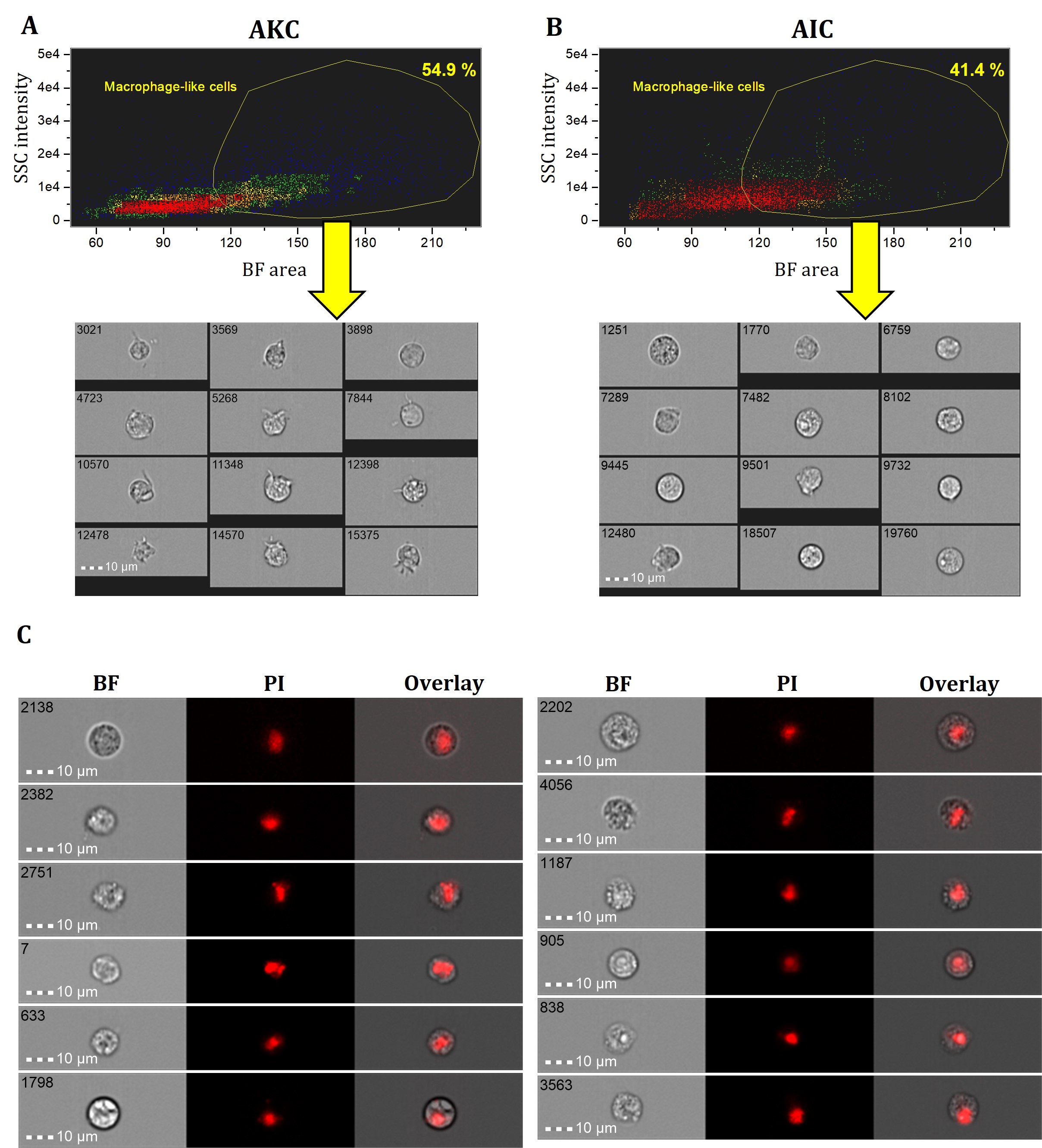

Supplement: Supplementary Figure 2 — Identification of macrophage-like cells in the AIC and AKC populations using imaging flow cytometry. Macrophage-like cells were identified based on the morphologies of cells and shapes of the nuclei. The areas of macrophage-like cells in AIC (A) and AKC (B) were gated in a bright field (BF) area (cell size) vs. side scatter (SSC) intensity (cell granularity) plot. Proportions of the macrophage-like cells among the whole adherent cell pool is indicated in the figure. Representative images from each gate are shown; (C) Propidium iodide (PI) staining revealed the shapes of the nuclei of intestinal macrophage-like cells in AIC (C). All cell images were captured with 40 × objective. Scale bar = 10 μm. [file Image_2.jpeg]

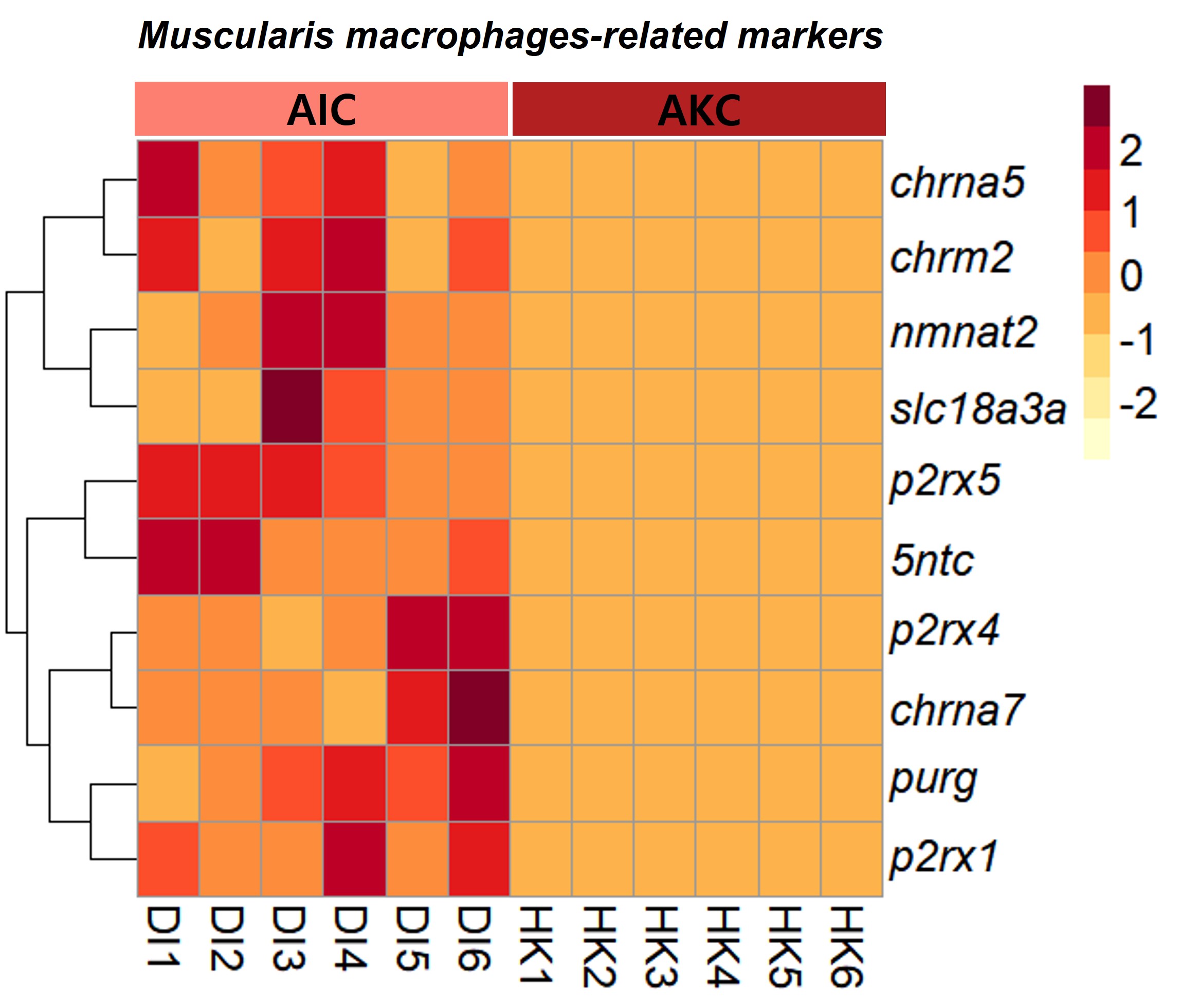

Supplement: Supplementary Figure 3 — Heatmap showing muscularis macrophage-related genes that were upregulated in adherent cells from the distal intestine of Atlantic salmon. AIC: adherent cells from the distal intestine; AKC: adherent cells from the head kidney; p2rx5: P2X purinoceptor 5-like; 5ntc: cytosolic purine 5-nucleotidase; p2rx4: P2X purinoceptor 4-like; purg: purine-rich element-binding protein gamma pseudogene; p2rx1: P2X purinoceptor 1-like; nmnat2: nicotinamide/nicotinic acid mononucleotide adenylyltransferase 2; chrna5: neuronal acetylcholine receptor subunit alpha-5-like; chrm2: muscarinic acetylcholine receptor M2-like; slc18a3a: probable vesicular acetylcholine transporter-A; chrna7: neuronal acetylcholine receptor subunit alpha-7-like. [file Image_3.jpeg]
